# Alterations in metabolome and lipidome in patients with in‐stent restenosis

**DOI:** 10.1111/cns.14832

**Published:** 2024-07-15

**Authors:** Ziqi Xu, Chenye Mou, Renjie Ji, Hanfen Chen, Yuge Ding, Xiaoyi Jiang, Fanxia Meng, Fangping He, Benyan Luo, Jie Yu

**Affiliations:** ^1^ Department of Neurology, First Affiliated Hospital, School of Medicine Zhejiang University Hangzhou China

**Keywords:** biomarkers, in‐stent restenosis, lipidomics, metabolomics, methionine

## Abstract

**Context:**

In‐stent restenosis (ISR) can lead to blood flow obstruction, insufficient blood supply to the brain, and may even result in serious complications such as stroke. Endothelial cell hyperproliferation and thrombosis are the primary etiologies, frequently resulting in alterations in intravascular metabolism. However, the metabolic changes related to this process are still undermined.

**Objective:**

We tried to characterize the serum metabolome of patients with ISR and those with non‐restenosis (NR) using metabolomics and lipidomics, exploring the key metabolic pathways of this pathological phenomenon.

**Results:**

We observed that the cysteine and methionine pathways, which are associated with cell growth and oxidative homeostasis, showed the greatest increase in the ISR group compared to the NR group. Within this pathway, the levels of N‐formyl‐l‐methionine and L‐methionine significantly increased in the ISR group, along with elevated levels of downstream metabolites such as 2‐ketobutyric acid, pyruvate, and taurocholate. Additionally, an increase in phosphatidylcholine (PC) and phosphatidylserine (PS), as well as a decrease in triacylglycerol in the ISR group, indicated active lipid metabolism in these patients, which could be a significant factor contributing to the recurrence of blood clots after stent placement. Importantly, phenol sulfate and PS(38:4) were identified as potential biomarkers for distinguishing ISR, with an area under the curve of more than 0.85.

**Conclusions:**

Our study revealed significant metabolic alterations in patients with ISR, particularly in the cysteine and methionine pathways, with phenol sulfate and PS(38:4) showing promise for ISR identification.

## Graphical abstract

Metabolomic and lipidomic analysis showed that patients who developed in‐stent restenosis (ISR) had significantly increased expression of L‐methionine and N‐formyl‐l‐methionine, and significantly decreased expression of triacylglycerol in their serum. Phenol sulfate and PS(38:4) may be potential biomarkers for the diagnosis of ISR.

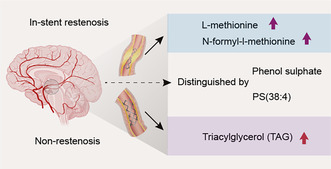

## 1 INTRODUCTION

In the last few decades, stroke has emerged as the leading cause of mortality in China and the second leading cause globally.^1^, ^2^ Prior research has demonstrated that the prevalence of intracranial atherosclerotic stenosis (ICAS) generally varies, constituting 10%–15% of ischemic stroke cases in Western countries and surging to 46.6% in Asia.^3^ Endovascular treatment and standard medical therapy are the mainstay of clinical treatment and can significantly reduce stroke recurrence rates in patients with ICAS.^4^ However, approximately 30% of patients may experience in‐stent restenosis (ISR),^5^ and the underlying mechanisms of this phenomenon remain undetermined. Interestingly, recent research on cardiovascular stent implantation has demonstrated a correlation between restenosis and changes in certain metabolites.^6^

Metabolites are the predominant biomolecules in the human body^7^ that are transported to various organs throughout the body via the bloodstream.^8^ Some metabolites enter tissue cells through transporters on endothelial cells or through endocytosis and exocytosis,^9^ while others are utilized by endothelial cells themselves to maintain normal physiological function.^10^ When blood vessels are damaged, endothelial cell dysfunction leads to abnormal absorption or release of metabolites, resulting in brain dysfunction.^11^ Restenosis can manifest within 1 year following stent intervention, primarily attributed to inflammatory response, which may activate the mTOR signaling pathway, leading to excessive proliferation of the local vascular endothelial cells.^12^ On the other hand, a study demonstrated that when patients accepted stent intervention, blood clots developed on the stent surface again, leading to vasoconstriction.^13^ Although rapamycin, an inhibitor of mTOR, can inhibit endothelial cell hyperproliferation,^14^ its upstream metabolic changes have yet to be ascertained. Therefore, gaining an understanding of differential metabolism under both ISR and non‐restenosis (NR) is crucial. This knowledge could ultimately pave the way for improving the mechanism of ISR.

Metabolomics and lipidomics represent advanced, high‐throughput methodologies designed for the systematic examination of comprehensive metabolic profiles.^15^ These techniques have demonstrated effectiveness in identifying alterations within lipid classes, including specific lipid species, and in predicting their potential roles in various biological processes.^16^ Notwithstanding their potential, the application of metabolomics and lipidomics in understanding the pathogenesis of arterial stenosis after stent intervention has rarely been investigated. In this study, we compared the global metabolic profile of serum between the patients with ISR and those without it, that is, NR. In addition, using metabolomics and lipidomics, we employ machine learning techniques to identify specific metabolites and accurately differentiate between patients with ISR and those without it, that is, NR.

## 2 METHODS

### 2.1 Subjects

Twenty‐one patients who underwent stent implantation after experiencing a transient ischemic attack or ischemic stroke were recruited from the Department of Neurology of the First Affiliated Hospital, Zhejiang University School of Medicine, between September 2020 and December 2021. Among these patients, the occurrence of ISR was observed in nine patients after intracranial stenting at the 1‐year follow‐up visit. ISR was defined as greater than 50% within or immediately adjacent (within 5 mm) to the stent implanted that was detected by digital subtraction angiography (DSA). Blood glucose and blood pressure values were in the normal range in all patients. All patients were receiving long‐term treatment with either atorvastatin or rosuvastatin. Patients with a history of inflammatory bowel diseases, hyperlipidemia, renal insufficiency, gastritis, or hepatitis were excluded.

### 2.2 lood sample collection

All fasting blood samples from patients who underwent stent implantation were collected into vacuum blood tubes. Then, the blood samples were centrifuged at 3000 rpm for 15 min at 4°C at once. The supernatants were collected to obtain serum samples. All samples were immediately stored at −80°C until metabolomics and lipidomics detection.

### 2.3 Pretreatment of serum samples for metabolite and lipid extraction

All serum samples were thawed at 4°C before pretreatment. The method for metabolite extraction has been described in previous studies.^17^, ^18^ First, 400 μL cold methanol was added to the 100 μL serum samples. Following thorough vortexing (Vortex‐Genie2, Scientific Industries, Bohemia, NY, USA), the samples were centrifuged at 12,000 rpm for 10 min at 4°C. The supernatants were transferred to the new 1.5‐mL centrifuge tubes. A concentrator (Genevac Ltd, Ipswich, Suffolk, UK) was used to remove solvents and concentrate samples. Finally, the samples were redissolved in 100 μL 1% (v/v) acetonitrile, and the supernatants were collected for further liquid chromatography‐coupled mass spectrometry (LC‐MS) analysis. Lipids were extracted according to the modified Bligh and Dyer's protocol as described in a previous study.^19^

### 2.4 Lipidomics analysis (LC‐MRM‐MS)

Lipid samples were separated using SCIEX Triple Quad 4500MD LC‐MS/MS System on a Phenomenex Luna silica column (3 μm, 2.0 × 150 mm). Mobile phase A contained chloroform‐methanol‐ammonia (89.5:10:0.5, v/v/v), and mobile phase B contained chloroform‐methanol‐ammonia (55:39:0.5:5.5, v/v/v). The gradient elution was 95% A for 5 min and was linearly decreased to 60% in 7 min and kept for 4 min. Then, 60% A was further decreased to 30% and kept for 15 min. Finally, a 5‐min re‐equilibration period using original gradient A was employed. The electrospray ionization (ESI) mode was used with the following parameters: curtain gas 20 psi; ion spray voltages 5500 V; temperature 400°C; ion source gases 1 and 2 with 35 psi. The multiple reaction monitoring (MRM) mode was set up for identification and quantitative analysis of various lipids. Lipid abundance was quantified by referencing the corresponding spiked internal standards.

### 2.5 Untargeted metabolomic analysis (UPLC–MS/MS)

Pretreated samples were separated by using ultraperformance liquid chromatography (Agilent 1290 II, Agilent Technologies, Germany) coupled to Quadrupole‐TOF MS (5600 Triple TOF Plus, Sciex, Singapore) on an ACQUITY UPLC HSS T3 column (1.8 μm, 3.0 × 100 mm, Waters, Dublin, Ireland). The details of LC‐MS/MS parameters have been described in a previous publication.^20^ Briefly, the LC mobile phase A consisted of water containing 0.1% formic acid (FA, buffer A), and mobile phase B was acetonitrile (ACN, buffer B). The linear gradient was used as follows: 2% B for 1 min; 2%–42% B during 1–6 min; 42%–65% B during 6–8 min; 65%–76% B during 6–10 min; 76%–100% B during 10–11 min and kept 100% B for 3 min. The following MS parameters for detection were set: curtain gas 35 psi; ion spray voltages 5500 V (positive‐ion mode) and −4500 V (negative‐ion mode); temperature 450°C; ion source gases 1 and 2 with 50 psi. The MS/MS analyses were performed in the information‐dependent acquisition mode with the collision energy set at 35 ± 15 eV. Data were acquired and processed using Analyst TF 1.7.1 software.

### 2.6 Data processing

A data matrix containing peak area, mass‐to‐charge ratio (*m/z*), and retention time extracted from the raw MS1 data was generated by using MarkerView version 1.3 (SCIEX, Concord, ON, Canada). The secondary identification of metabolites was based on databases Metabolites, HMDB, METIN, and standard references by MS2 data using PeakView version 2.2 (SCIEX, Concord, ON, Canada). The identified metabolites were matched to the corresponding ion in the MS1 data matrix. Then, the data were further analyzed using self‐programmed statistical and pathway analysis based on R.

### 2.7 Pattern recognition analysis

Original data were normalized by dividing by the sum of the concentration of each lipid or metabolite. The processed data were uploaded to MetaboAnalyst version 5.0 (www.metaboanalyst.ca) for further analysis.^18^, ^21^ After logistic transformation and auto‐scaling, principal component analysis (PCA) and partial least squares discriminant analysis (PLS‐DA) models were established. The variable importance in the projection (VIP) representing the contribution of each variable to the classification was calculated in the PLS‐DA model. The univariate analysis included Student's *t*‐test and fold‐change analysis of variables.

### 2.8 Statistical analysis

Statistical analyses were conducted using MetaboAnalyst version 5.0 and Prism 8.0.2. (GraphPad Software Inc., San Diego, CA, USA). Continuous variables were expressed as mean ± standard error of the mean (SEM), mean ± standard deviation (SD), or median (25th–75th percentile) when appropriate. Categorical variables were expressed as numbers (%) and were compared using the Fisher exact test. PCA, dendrogram, volcano plot, and heatmap analyses were performed using R software version 4.3.1 to screen for differential metabolites and lipids. Pathway analysis is based on the Kyoto Encyclopedia of Genes and Genomes (KEGG) database. *T*‐tests or nonparametric Mann–Whitney tests were conducted to evaluate the variances in lipid or metabolite expression based on the data distribution. Pearson's correlation analysis and single‐factor logistic regression analysis were used to identify potential biomarkers. Elastic net regression, a machine learning algorithm, was employed in conjunction with a nested cross‐validation procedure based on R packages *glmnet*, *caret*, and *nestedcv* in order to screen for multivariable metabolites. The 10‐fold cross‐validation (CV) was selected as the inner fold to optimize the hyperparameters *α* and the optimal *λ* for the model. The leave‐one‐out cross‐validation (LOOCV) was selected as the outer folds for model validation. A receiver operating characteristic (ROC) curve analysis was conducted to evaluate the efficacy of the model. A *p*‐value <0.05 was deemed statistically significant.

## 3 RESULTS

Twenty‐one patients who underwent stent implantation between September 2020 and December 2021 were enrolled. Of these, nine patients developed ISR, as assessed by digital subtraction angiography (DSA) at 12‐month follow‐up. Clinical and demographic characteristics are encapsulated in Table 1. Consistent with the results of previous research, our results revealed no significant differences in the laboratory results between NR and ISR patients.^22^ The characteristics of the stent, such as diameter, length, and type, as well as the location of the lesion, were comparable between patients with NR and ISR.^23^

**TABLE 1:** Baseline characteristics of patients.

	NR (*n* = 12)	ISR (*n* = 9)	*p*
Age, mean (SD), y	59.4 ± 11.8	57.8 ± 12.3	0.76
BMI (kg/m^2^)	24.6 ± 2.7	25.3 ± 3.5	0.60
Male	9 (75.0)	5 (55.6)	0.40
Medical history			
Hypertension (%)	8 (66.7)	5 (55.6)	>0.99
Smoking (%)	3 (25.0)	2 (22.2)	>0.99
Drinking (%)	2 (22.2)	1 (11.1)	>0.99
Hyperlipidemia (%)	0 (0.0)	0 (0.0)	>0.99
Coronary artery disease (%)	0 (0.0)	0 (0.0)	>0.99
Laboratory results			
CRP (mg/L)	3.6 ± 5.1	2.8 ± 3.3	0.66
PLT (× 10^9^/L)	207.9 ± 33.6	224.2 ± 35.5	0.30
HbA1_C_ (%)	6.7 ± 1.0	6.8 ± 1.2	0.89
Fibrinogen (g/L)	2.7 ± 0.6	2.9 ± 0.6	0.56
eGFR (ml/min/1.73 m^2^)	94.9 ± 17.7	91.4 ± 13.0	0.63
Hcy (μmol/L)	10.9 ± 3.2	10.5 ± 3.6	0.77
Free fatty acid (mmol/L)	0.5 ± 0.2	0.6 ± 0.2	0.23
Uric acid (μmol/L)	282.1 ± 130.1	315.6 ± 102.1	0.53
Cholesterol (mmol/L)	3.6 ± 1.0	3.7 ± 0.9	0.87
LDL‐C (mmol/L)	1.8 ± 0.6	3.1 ± 3.4	0.19
Lipid‐lowering drugs			
Atorvastatin (%)	10 (83.3)	8 (88.9)	‐
Rosuvastatin (%)	2 (16.7)	1 (11.1)	‐
Stent information			
Stent diameter (mm)	3.5 ± 0.9	3.5 ± 0.7	>0.99
Stent length (mm)	17.4 ± 6.0	18.3 ± 8.3	0.80
Stent type			
Wingspan (%)	3 (25.0)	3 (33.3)	>0.99
APOLLO (%)	2 (16.7)	0 (0.0)	0.49
Enterprise (%)	2 (16.7)	2 (22.2)	>0.99
Neuroform EZ (%)	5 (41.7)	4 (44.4)	>0.99
Culprit vessel			
L/RM1 (%)	8 (66.7)	6 (66.7)	‐
L/RV4 (%)	2 (16.7)	1 (11.1)	‐
L/RC6 (%)	2 (16.7)	1 (11.1)	‐
BA (%)	0 (66.7)	1 (11.1)	‐
% Stenosis, median [IQR]	77.3 [73.7–95.3]	85.0 [65.6–100.0]	0.39
% Restenosis, median [IQR]	33.5 [11.4–37.3]	60.0 [55.4–91.2]	<0.001

*Note*: Values are presented as mean ± standard deviation (SD), numbers (%), or median [25th–75th percentile]. *p* values were calculated by using *t*‐test or Fisher exact test, where appropriate.

Abbreviations: BA, basilar artery; BMI, body mass index; CRP, C‐reactive protein; eGFR, estimated glomerular filtration rate; HbA1C, hemoglobin A1c; Hcy, homocysteine; IQR, interquartile range; ISR, in‐stent restenosis; LDL‐C, low‐density lipoprotein cholesterol; L/RC6, left/right carotid artery 6 segments; L/RM1, left/right middle cerebral artery M1 segment; L/RV4, left/right vertebral artery 4 segments; NR, non‐restenosis; PLT, platelet count.

### 3.1 Untargeted metabolomic analysis of serum from NR and ISR patients

**FIGURE 1:**
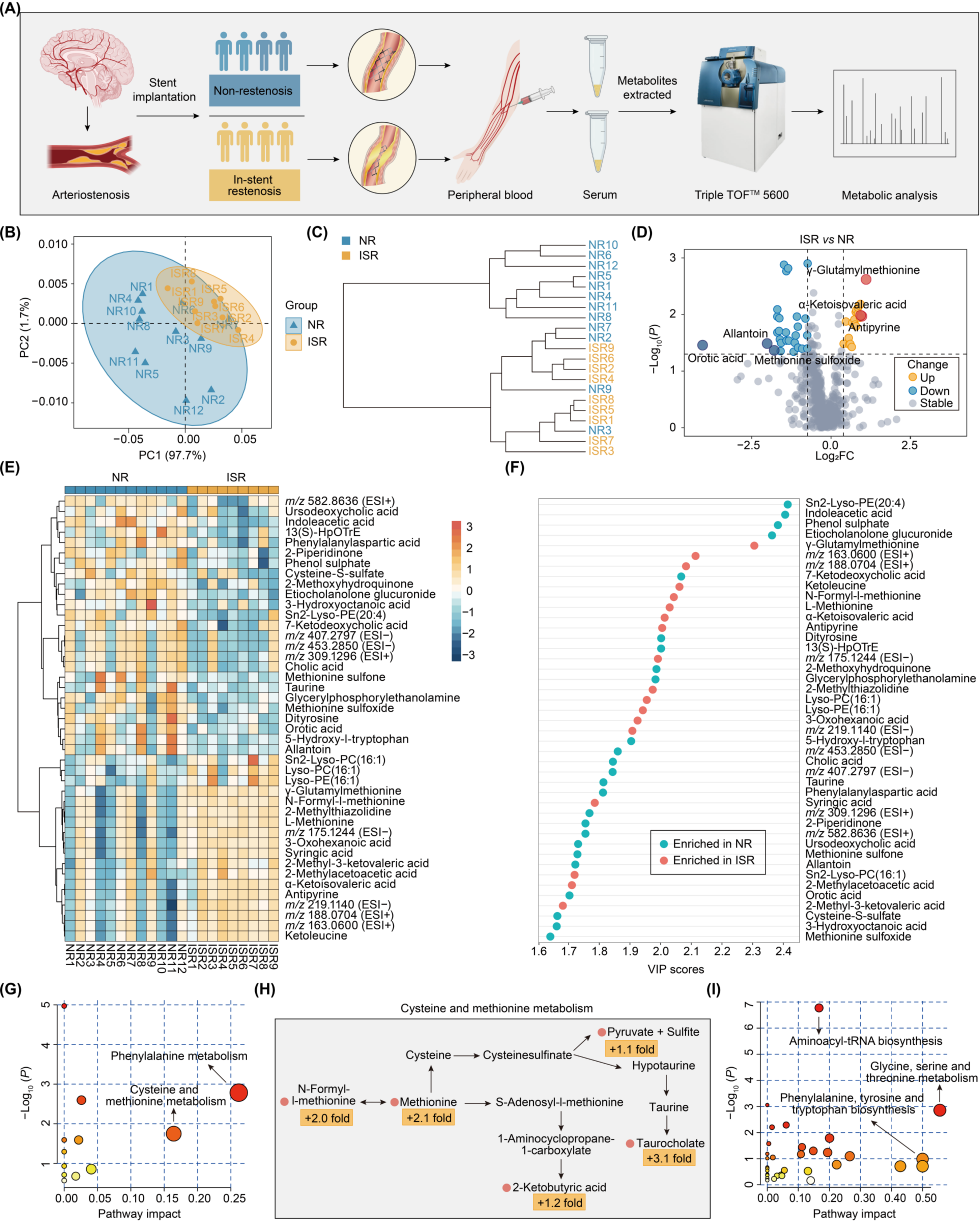
The metabolomes of serum from NR and ISR groups based on untargeted metabolomics. (A) Flowchart of nontargeted analyses. Flowchart outlining the process of processing serum samples for metabolomics. (B) Principal component analysis (PCA) of the metabolomes of serum from NR and ISR patients. Two PCs explained 99.4% (97.7% and 1.7%) of the variance between NR and ISR patients. PC scores are indicated as %; triangle indicate individual samples from the two groups. (C) Dendrogram showing the clustering of all 21 samples. (D) Volcano plot differential metabolites between NR and ISR groups. Differential (yellow or blue) and nondifferential (gray) metabolites were defined by the criteria of fold change >1.5 and *p* < 0.05. A data point in the negative values of the fold change represents a high level of a metabolite in the NR group, and a data point in the positive values represents a high level of a metabolite in ISR group. Top 3 upregulated (red) or downregulated metabolites (dark blue) in ISR group are labeled. (E) Heatmap representation of 43 of 82 metabolites in the two groups, as identified by fold change >1.5 and *p* < 0.05. Each column represents a sample from NR group or ISR group. Orange indicates a greater abundance of the metabolite. (F) Top 43 metabolites based on VIP scores for differentiating between NR group and ISR group. The colored boxes on the left indicate the relative concentrations of the corresponding metabolites averaged across each group. VIP, variable importance in the projection. (G) Pathway analysis using metabolites upregulated in the ISR patients. The size of the circles depends on the counts of metabolites enriched in the pathway. (H) A summary of cysteine and methionine metabolism pathway, with the metabolites upregulated in the ISR patients labeled in yellow. (I) Pathway analysis using metabolites downregulated in the ISR patients. ISR, in‐stent restenosis; NR, non‐restenosis.

To determine the metabolic difference between NR and ISR patients, fasting serum samples were collected from the patients for untargeted metabolomics analysis. The workflow is presented in Figure 1A. A total of 769 metabolite features were detected, and 481 of them were annotated from four databases, namely, HMDB, PubChem, METLIN, and Lipid Maps. By establishing a PCA model based on the peak intensity of the metabolites, we found that the 21 samples could be divided into two groups (Figure 1B). Similar results were obtained via cluster analysis (Figure 1C). To further investigate which of the metabolites contribute to the differences, a volcano analysis was performed (Figure 1D). With the condition of fold change (FC >1.5 and *p* < 0.05), the significantly differential metabolites, such as γ‐glutamylmethionine, N‐formyl‐l‐methionine, and L‐methionine, were increased in the ISR group, while orotic acid, allantoin, and methionine sulfoxide were increased in the NR group (Figure 1D). These results suggested that patients with ISR may have dysfunction in the metabolism of methionine, which is a sulfur‐containing essential amino acid associated with oxidative stress.^24^ Furthermore, orotic acid and allantoin have been reported to exhibit neuroprotective function.^25^, ^26^ The decreased concentration of these metabolites could potentially contribute to the development of ISR. We further performed PLS‐DA to identify the metabolites that maximized the separation between the two groups. We identified 43 metabolites that maximized the separation based on the VIP score. Among these metabolites, γ‐glutamylmethionine, N‐formyl‐l‐methionine, and L‐methionine were enriched in the ISR group, while sn2‐Lyso‐PE(20:4), indoleacetic acid, and phenol sulfate were enriched in the NR group (Figure 1E,F).

To investigate the up and downregulated pathways in the ISR group compared with the NR group, a comprehensive global pathway analysis was performed using the significantly differential metabolites (FC >1.5, *p* < 0.05, and VIP >1.6), with the KEGG database serving as the reference. The phenylalanine metabolism pathway was the most significantly upregulated in ISR patients compared with NR patients (Figure 1G). The intermediate metabolites of phenylalanine were suggested to promote the release of inflammatory factors such as IL‐1β and IL‐6, increasing the risk of cardiovascular events,^27^ which may be one of the primary reasons for stimulating the excessive proliferation of endothelial cells. Another significant metabolic pathway, the cysteine and methionine pathway, which is related to oxidative stress and endothelial dysfunction, was also significantly increased in ISR patients.^24^, ^28^ Of these pathways, the relative abundances of the key metabolites, such as N‐formyl‐l‐methionine, methionine, 2‐ketobutyric acid, pyruvate, and taurocholate, were markedly increased (Figure 1H), indicating that they might play a particular role in the pathology of the occurrence of ISR. In contrast, the aminoacyl‐tRNA biosynthesis pathway, glycine, serine, and threonine metabolism pathway, and phenylalanine, tyrosine, and tryptophan biosynthesis pathway were the most significantly downregulated pathways in ISR patients compared with that in NR patients (Figure 1I).

### 3.2 Comparison of metabolite abundance between NR and ISR patients

**FIGURE 2:**
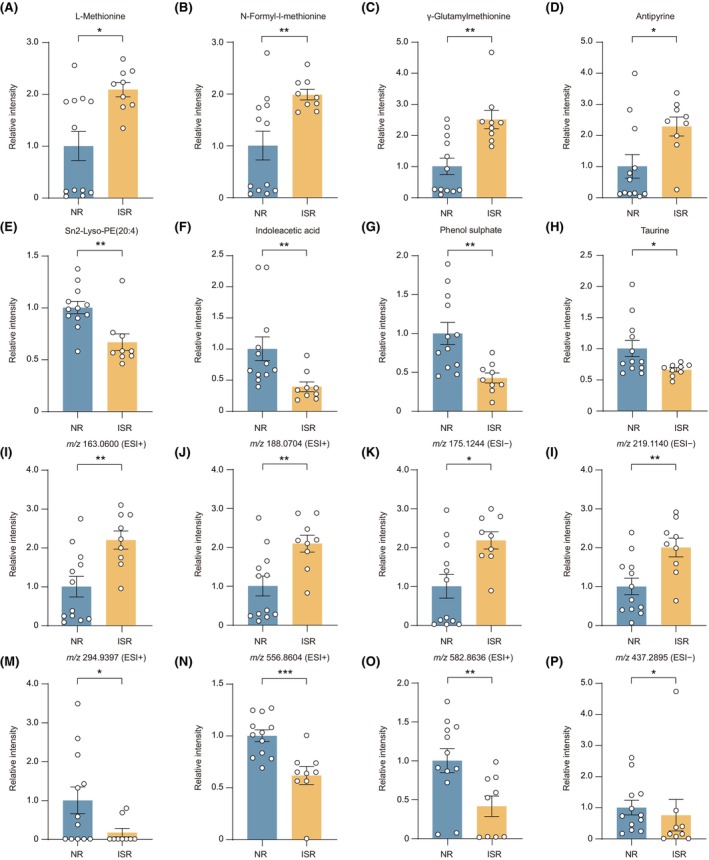
Representative differential metabolites between NR and ISR groups. (A–H) Relative peak intensities of representative identified metabolites between NR and ISR groups from serum, including L‐Methionine (A), N‐Formyl‐l‐methionine (B), γ‐Glutamylmethionine (C) Antipyrine (D), sn2‐Lyso‐PE(20:4) (E), Indoleacetic acid (F), Phenol sulfate (G), Taurine (H). (I–P) Relative peak intensities of representative unidentified metabolites between NR and ISR groups from serum, including *m/z* 163.0600 (I), *m/z* 188.0704 (J), *m/z* 175.1244 (K), *m/z* 219.1140 (L), *m/z* 294.9397 (M), *m/z* 556.8604 (N), *m/z* 582.8636 (O), *m/z* 437.2895 (P). Data represent the mean ± SEM; *p < 0.05, **p < 0.01, ***p < 0.001, Wilcox nonparametric test. ISR, in‐stent restenosis; ESI (+), electrospray ionization positive mode; ESI (−), electrospray ionization negative mode; *m/z*, mass to charge ratio; NR, non‐restenosis.

To determine the metabolites associated with ISR development, we performed an unpaired comparison analysis. The identified metabolites we observed with the greatest increase in ISR patients were L‐methionine, N‐formyl‐l‐methionine, γ‐glutamylmethionine, and antipyrine (Figure 2A–D), whereas sn2‐Lyso‐PE(20:4), indoleacetic acid, phenol sulfate, and taurine were most reduced (Figure 2E–H). We also found several important unidentified metabolites enriched in the ISR group, as indicated by their mass‐to‐charge ratio (*m/z*). The four most highly up or downregulated metabolites were displayed, which were *m/z* 163.0600, *m/z* 188.0704, *m/z* 175.1244, *m/z* 219.1140, *m/z* 294.9397, *m/z* 556.8604, *m/z* 582.8636, and *m/z* 437.2895 (Figure 2I–P). These metabolites are crucial for ISR patients and need to be further identified in the future.

### 3.3 Lipidomic analysis distinguishes ISR patients from NR patients

**FIGURE 3:**
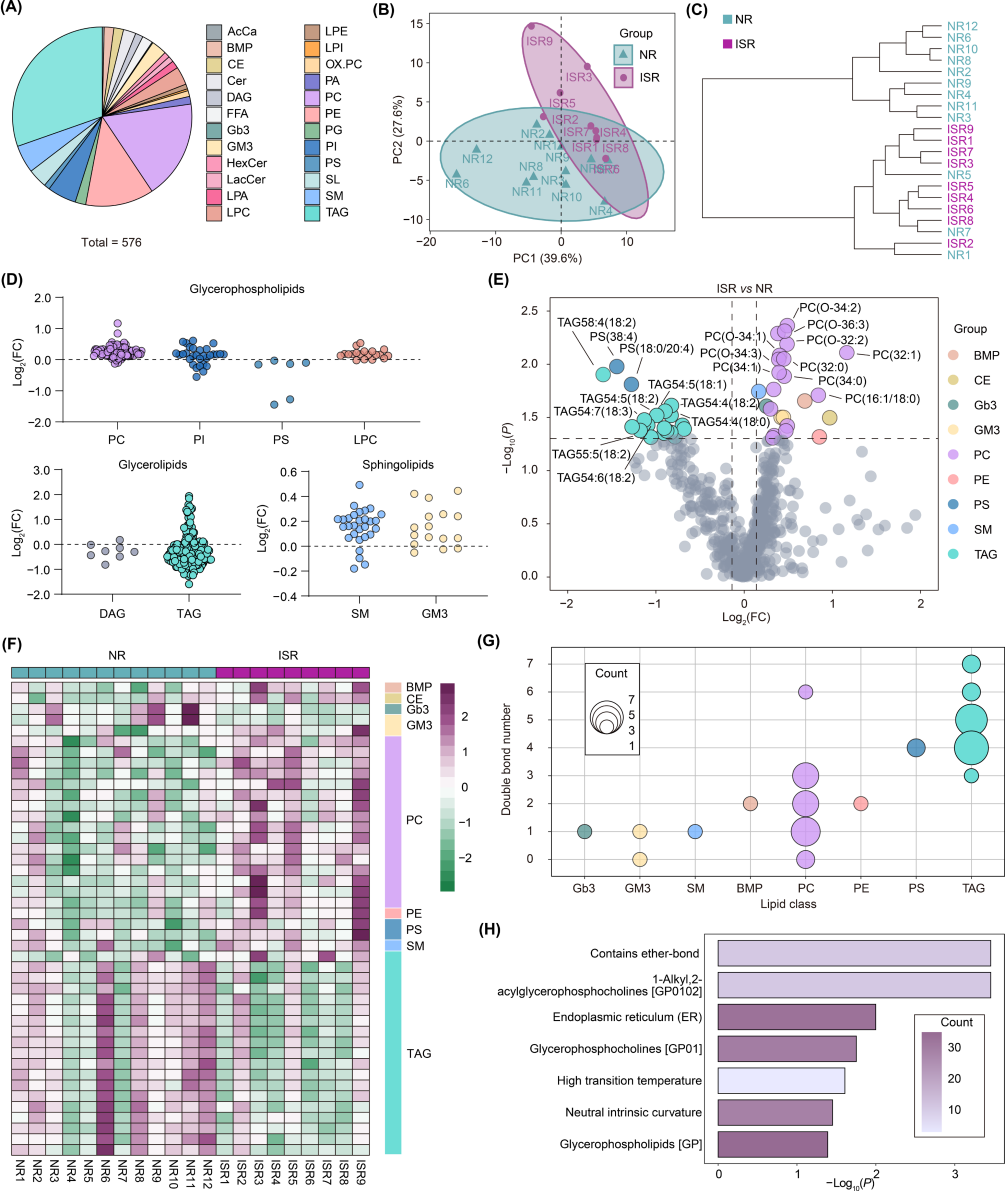
The lipid profiling of serum from NR and ISR groups based on lipidomics. (A) Pie chart for distribution of a total of 576 metabolites, including 24 subclasses. (B) Principal component analysis (PCA) of the metabolomes of serum from NR and ISR patients. Two PCs explained 67.2% (39.6% and 27.6%) of the variance between NR and ISR patients. PC scores are indicated as %; triangle indicate individual samples from the two groups. (C) Dendrogram of the NR and ISR groups showing the clustering of all 21 samples. (D) Dot plot of the abundance of lipids grouped by subclasses from ISR group relative to NR group. Each dot represents one kind of lipid. The dotted line indicates equal abundance of lipid in the NR and ISR groups. (E) Volcano plot showing the distribution of statistically enriched (FC >1.1, *p* < 0.05) lipids. Top 10 most significantly changed lipids are labeled. (F) Heatmap with clustering of normalized lipid concentration for significantly changed lipids (*p* < 0.05). Each column represents a sample from NR group or ISR group. Purple indicates a greater abundance of the lipids. (G) Bubble plot showing the distribution of double bond number of lipids grouped by lipid class. The size of the bubble indicates the counts of lipids. (H) Lipid ontology analysis for significantly altered lipids (*p* < 0.05) from ISR group relative to NR group. FC, fold change; ISR, in‐stent restenosis; NR, non‐restenosis.

In addition to the polar metabolites we detected, such as amino acids, nucleotides, and/or vitamins, lipids account for 85% of the total metabolites in the brain and play a key role in cerebrovascular diseases.^29^, ^30^ Dyslipidemia has been extensively reported as a risk factor for ICAS in prior research.^31^ We found a tendency for LDC to be relatively elevated in the ISR group Therefore, we further performed targeted lipidomics to investigate the lipid metabolism of the same patients. A total of 576 lipids in 24 subclasses were identified (Figure 3A), including acylcarnitine (AcCa), bis(monoacylglycero)phosphate (BMP), cholesteryl ester (CE), ceramide (Cer), diacylglycerol (DAG), free fatty acids (FFA), globotriaosylceramide (Gb3), monosialodihexosylganglioside (GM3), hexosylceramide (HexCer), lactosylceramide (LacCer), lyso‐phosphatidic acid (LPA), lyso‐phosphatidylcholine (LPC), lyso‐phosphatidylethanolamine (LPE), lyso‐phosphatidylinositol (LPI), oxidized phosphatidylcholine (OX.PC), phosphatidic acid (PA), phosphatidylcholine (PC), phosphatidylethanolamine (PE), phosphatidylglycerol (PG), phosphatidylinositol (PI), phosphatidylserine (PS), sphingolipids (SL), sphingomyelin (SM), and triacylglycerol (TAG). Similar to the findings from the metabolomics, the 21 samples were categorized into two groups according to lipid concentration via the PCA model (Figure 3B). Using cluster analysis, we also found that samples from NR and ISR patients could be easily distinguished (Figure 3C). In subclass comparison, we detected no significant changes in lipid subclass composition between the two groups of patients. However, the majority of lipids in TAG and DAG, which are classified as glycerolipids, showed a decrease in ISR patients compared with NR patients (Figure 3D). Meanwhile, the majority of lipids in PC, PI, and LPC, classified as glycerophospholipids, and SM and GM3, classified as sphingolipids, showed an increase in ISR patients compared with NR patients (Figure 3D). Using volcano analysis, we identified 24 lipids that exhibited significant changes (FC >1.1 and *p* < 0.05) in patients with ISR (Figure 3E). Most of the increased lipids were PC, such as PC(32:1), PC(O‐34:2), and PC(O‐36:3), while most of the decreased lipids were TAG, such as TAG58:4(18:2), TAG54:5(18:1), and TAG54:7(18:3) (Figure 3E). The lipid subclass‐ordered heatmap also discriminated between ISR and NR patients (Figure 3F). Additionally, fatty acyl side chain composition and degree of unsaturation play a vital role in determining lipid biophysical properties. Interestingly, most of the significantly differential lipids carry unsaturated fatty acid chains, such as PC, PE, and SM, involved in the synthesis and stability of cell membranes (Figure 3G). Furthermore, we conducted a lipid ontology enrichment analysis. The results revealed that lipids significantly increased in ISR patients and were significantly enriched in the endoplasmic reticulum (ER) and glycerophosphocholines (Figure 3H). These changes may relate to the abnormal proliferation of endothelial cells in ISR patients.

### 3.4 Comparison of lipid abundance between NR and ISR patients

**FIGURE 4:**
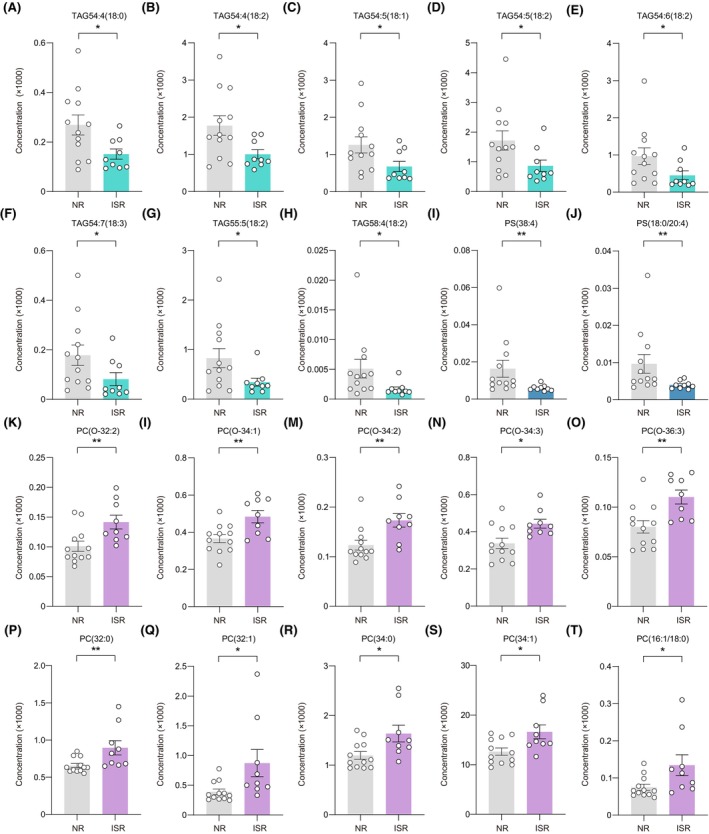
Representative differential lipids between NR and ISR groups. (A–J) Representative lipids downregulated in the ISR patients from serum, including TAG54:4(18:0) (A), TAG54:4(18:2) (B), TAG54:5(18:1) (C), TAG54:5(18:2) (D), TAG54:6(18:2) (E), TAG54:7(18:3) (F), TAG55:5(18:2) (G), TAG58:4(18:2) (H), PS(38:4) (I), PS(18:0/20:4) (J). (K–T) Representative lipids upregulated in the ISR groups from serum, including, PC(O‐32:2) (K), PC(O‐34:1) (L), PC(O‐34:2) (M), PC(O‐34:3) (N), PC(O‐36:3) (O), PC(32:0) (P), PC(32:1) (Q), PC(34:0) (R), PC(34:1) (S), and PC(16:1/18:0) (T). Data represent the mean ± SEM; *p < 0.05, **p < 0.01, Wilcox nonparametric test. ISR, in‐stent restenosis; *m/z*, mass to charge ratio; NR, non‐restenosis; PC, phosphatidylcholine; PS, phosphatidylserine; TAG, triacylglycerols.

We noted the lipids with the greatest changes in up and downregulation between ISR and NR patients. Of the downregulated lipids, eight belonged to TAG, including TAG54:4(18:0), TAG54:4(18:2), TAG54:5(18:1), TAG54:5(18:2), TAG54:6(18:2), TAG54:7(18:3), TAG55:5(18:2), and TAG58:4(18:2), while the other two belonged to PS, including PS(38:4) and PS(18:0/20:4) (Figure 4A–J). Notably, all elevated lipids were from PC, including PC(O‐32:2), PC(O‐34:1), PC(O‐34:2), PC(O‐34:3), PC(O‐36:3), PC(32:0), PC(32:1), PC(34:0), PC(34:1), and PC(16:1/18:0) (Figure 4K–T).

### 3.5 Identification of potential blood biomarkers for the prediction of ISR

**FIGURE 5:**
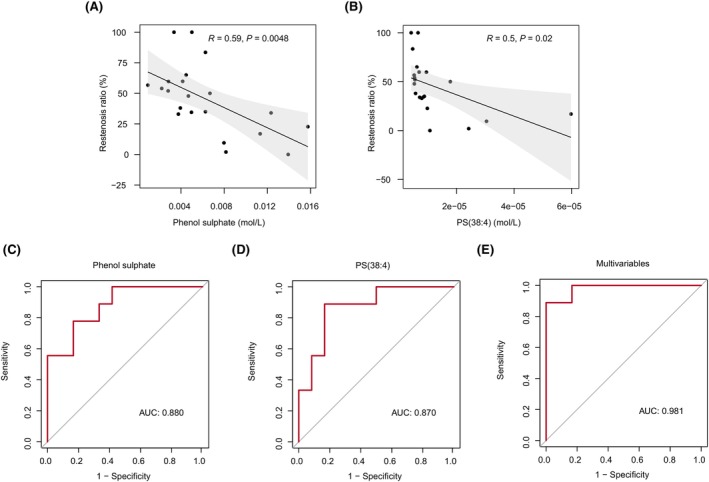
Identification of candidate biomarkers for distinguishing ISR. (A, B) Pearson correlation analysis was performed using the concentration of phenol sulfate (A) or PS(38:4) (B) from the serum of the NR and ISR patients and their respective stenosis ratio. Calculated 95% confidence intervals are shown in gray. (C, D) The efficacy of phenol sulfate (C) or PS(38:4) (D) in distinguishing ISR patients from NR patients was determined by ROC analysis. (E) The efficacy of multivariables selected by elastic net regression in distinguishing ISR patients from NR patients was determined by ROC analysis. AUC, area under the ROC curve; ISR, in‐stent restenosis; NR, non‐restenosis; ROC, Receiver‐operating characteristic.

In our results, 16 metabolites were identified with significantly different levels between ISR and NR patients (FC >1.5, *p* < 0.05, and VIP >1.6). With the help of lipidomics, we also identified 20 lipids whose levels were significantly different between the two groups (FC >1.1, *p* < 0.05, and VIP >1.4). We first performed a correlation analysis between the concentration of metabolites and lipids and the restenosis rate to assess their potential as biomarkers to discriminate between ISR and NR patients. Finally, the levels of phenol sulfate, PS(38:4), and the rate of restenosis displayed a significant negative correlation (Figure 5A,B). To further evaluate their diagnostic efficacy, we performed a logistic regression analysis and conducted an ROC curve analysis to evaluate the performance of the model. Both phenol sulfate and PS(38:4) demonstrated high accuracy, achieving an area under the curve (AUC) of 0.880 and 0.870, respectively (Figure 5C,D). For phenol sulfate, the highest Youden's index was calculated at a cutoff of 0.0046 (Youden's index 0.61, sensitivity 83.33%, and specificity 77.78%). For PS(38:4), the highest Youden's index was calculated at a cutoff of 7.04 e^−06^ (Youden's index 0.72, sensitivity 88.89%, and specificity 83.33%). We developed an elastic net regression model to identify multiple metabolites that can predict ISR events effectively. The elastic net model combines the strengths of LASSO and Ridge regression techniques. It leverages the feature selection capability of LASSO while incorporating the Ridge penalty to address multicollinearity issues, enhancing the model's robustness and predictive power.^32^ A total of 36 variables with non‐zero coefficients were selected for model construction, with hyperparameters α set to 0.7 and λ set to 0.003239 (Supplementary Table S1). The ROC curve analysis illustrated strong predictive performance for ISR using these multivariable metabolic features, yielding an impressive AUC value of 0.981 (Figure 5E).

## 4 DISCUSSION

To the best of our knowledge, this study is the first to demonstrate an integrated metabolomics and lipidomics analysis performed to investigate the profiles of metabolites and lipids in the serum of NR and ISR patients. The metabolomic analysis revealed a significant increase in metabolites enriched in the cysteine and methionine pathways in patients with ISR. The lipidomic analysis revealed that glycerophospholipids, mainly PC, were elevated in ISR patients, while glycerolipids, especially TAG, were reduced in ISR patients. This discovery serves as a crucial point of reference for elucidating the metabolic mechanism of ISR. Our most significant discovery involved the identification of metabolites such as phenol sulfate and PS (38:4) as potential biomarkers with high accuracy for predicting ISR.

We noted that some significantly increased metabolites and metabolic pathways were enriched in ISR patients, especially the cysteine and methionine metabolism pathways. Cysteine and methionine are both sulfur‐containing amino acids.^33^ Methionine is an essential amino acid that plays a critical role in the initiation of translation and is a precursor to cysteine, glutathione, and S‐adenosylmethionine.^34^, ^35^, ^36^ Overloaded methionine may lead to endothelial cell dysfunction and increase the risk of cerebrovascular disease.^34^ In addition, the level of N‐formyl‐l‐methionine (fMet) increases along with the elevation of methionine in the serum of ISR patients. fMet was first discovered during the translation process in bacteria, chloroplasts, and mitochondria.^37^ It has also been identified as a potential predictive factor for coronary heart disease.^38^ Certain pathological states, such as an overload of reactive oxygen species, degradation of mitochondria, and abnormal energy metabolism disorders, can lead to an increased release of fMet and methionine from the brain.^39^ Furthermore, different degrees of damage to endothelial cells can further inactivate methionine‐related transporters, such as L‐type amino acid transporter 1 (LAT1), further leading to an overload of free methionine and fMet in the serum.^40^ Interestingly, we found that the abundance of taurine was significantly reduced in ISR patients, along with increased production of its downstream product, taurocholate. As one of the most abundant free amino acids in the human body, taurine plays various biological roles in the central nervous system,^41^ such as maintaining intra‐mitochondrial calcium homeostasis, modulating endoplasmic reticulum stress, and protecting against oxidative stress and neuroinflammation.^42^ Therefore, supplementing taurine may improve the function of endothelial cells and reduce oxidative stress to slow the progression of ISR.

In our results, most of the significantly increased serum lipids in ISR patients belonged to glycerophospholipids and sphingolipids, which are the main components of the membrane.^43^ Among glycerophospholipids, we observed that the subclass PC was the most significantly increased in ISR patients, which was related to endothelial cell proliferation,^44^ and it could promote cholesterol deposition.^45^ Of these, PC(O‐32:2), PC(32:0), PC(34:0), and PC(34:1) showed dramatically higher levels in ISR patients compared with NR patients and need to be further investigated. On the other hand, intimal hyperplasia and inflammation are the chief reasons for ISR.^46^ It has been reported that the metabolism of glycerophospholipids is a critical pathway involved in regulating the body's inflammatory state.^47^ The elevated level of serum PC may act as a potential inflammatory mediator by disrupting cell membrane stability and exacerbating the inflammatory response in patients with ISR. Moreover, the poly‐unsaturated fatty acid side chains were observed in most of the significantly changed lipids in ISR patients. Excessive unsaturated fatty acid chains may interfere with the utilization of other lipids in the brain, which may also be one of the reasons for endothelial cell hyperplasia.^48^ Contrarily, the level of other lipids, such as TAG and DAG, was significantly decreased in ISR patients. This may indicate that these patients have a more active lipid metabolism to achieve the increased efflux of these two categories.

The prediction and diagnosis of ISR are the most notable challenges in this field.^49^ At present, the clinical diagnosis mainly depends on carotid and intracranial artery ultrasound, CTA, and DSA.^50^ However, these methods have the characteristics of low accuracy, invasiveness, and large radiation.^51^ Based on the significant changes in the metabolic and lipidomic profiles, we found that phenol sulfate and PS(38:4) may help diagnose ISR. Low serum levels of phenol sulfate and PS(38:4) are associated with a high restenosis rate and may also be used as a potential biomarker for ISR with high accuracy.

Our study has a few limitations that should be addressed. First, although we did our best to exclude the interference of other factors, the effect of dairy intake on serum metabolomic and lipidomic analysis is not controllable. Second, the gender dimorphism of this study may have been overlooked in this study. A growing body of research has highlighted that women's vascular regulatory capacity diminishes with age, and women tend to have a poorer prognosis for stroke compared to men.^52^, ^53^, ^54^, ^55^ While our study did not reveal distinct gender differences between the ISR and NR groups, the potential impact of sex dimorphism on outcomes cannot be discounted. Further investigations focusing on gender‐specific experiments are warranted to deepen our understanding in this area. Third, as a retrospective study, we only collected the serum of patients at the 1‐year follow‐up. Further multicenter prospective studies and large sample sizes of cohorts are required.

## 5 CONCLUSIONS

In summary, we investigated the change in serum metabolism and lipids between NR and ISR patients and found some metabolites and lipids related to the mechanism of pathological changes in ISR. The abnormal cysteine and methionine metabolism pathway and glycerophospholipid levels may be the key factor for these pathological processes. We also identified phenol sulfate and PS(38:4) as the potential biomarkers for diagnosing patients with restenosis 1year after stent implantation.

## AUTHOR CONTRIBUTIONS

Jie Yu and Benyan Luo conceived and supervised the project. Ziqi Xu, Renjie Ji, Hanfeng Chen, and Jie Yu designed the experiments. Yuge Ding, Fangping He, and Fanxia Meng collected the serum samples. Xiaoyi Jiang and Fanxia Meng extracted metabolites. Ziqi Xu and Chenye Mou performed the untargeted metabolomics and targeted lipidomics. Jie Yu, Ziqi Xu, and Chenye Mou completed untargeted metabolomics and lipidomics analysis. Fangping He and Benyan Luo provided reagents. Chenye Mou and Jie Yu prepared figures. Ziqi Xu, Chenye Mou, and Jie Yu wrote the manuscript. All authors discussed, reviewed, and edited the manuscript.

## CONFLICT OF INTEREST STATEMENT

All authors declare no conflicts of interest.

## Supporting information


Supplementary Table S1.

## Data Availability

The data that support the findings of this study are available from the corresponding author upon reasonable request.
