# Supplementary material for: Alterations in metabolome and lipidome in patients with in‐stent restenosis
Source: CNS Neurosci Ther. 2024 Jul 15;30(7):e14832. doi: 10.1111/cns.14832 (PMC11249805; doi:10.1111/cns.14832)
Supplement: Supplementary file 1 — Supplementary Table S1. [file CNS-30-e14832-s001.docx]

**Supplementary Table 1**

| **Supplementary Table 1. Variables with non-zero coefficients** | | | | | | |
| --- | --- | --- | --- | --- | --- | --- |
|  | **Coefficients** | | |  | **Coefficients** | |
| γ-Glutamylmethionine | | 0.5956 | Indoleacetic acid | | | −0.9795 |
| Lyso-PC(14:0) | | 0.5234 | Sn2-Lyso-PE(20:4) | | | −0.8905 |
| Lyso-PC(16:1) | | 0.3701 | *m/z* 582.8636 (ESI+) | | | −0.7930 |
| PC(O-36:3) | | 0.3184 | Etiocholanolone glucuronide | | | −0.7166 |
| N-γ-Glutamylglutamine | | 0.3066 | Phenol sulphate | | | −0.6250 |
| PC(O-16:1/18:1) | | 0.2780 | Cysteine-S-sulfate | | | −0.4334 |
| *m/z* 163.0600 (ESI+) | | 0.2642 | TAG52:4(18:2) | | | −0.3783 |
| γ-Glutamylglutamine | | 0.2353 | Glycerylphosphorylethanolamine | | | −0.3249 |
| SM(d18:1/16:0) | | 0.2273 | 2-Piperidinone | | | −0.2475 |
| PC(O-34:3) | | 0.1774 | 2-Methoxyhydroquinone | | | −0.2145 |
| PC(O-34:1) | | 0.1743 | TAG55:4(18:2) | | | −0.1594 |
| N-Methyl-1H-indole-3-propanamide | | 0.1433 | TAG54:4(18:0) | | | −0.1273 |
| *m/z* 188.0704 (ESI+) | | 0.1170 | 2-Ethyl-2-hydroxybutyric acid | | | −0.0809 |
| PC32:2(16:1/16:1) | | 0.0908 | TAG55:5(18:2) | | | −0.0317 |
| GM3(d18:0/20:0) | | 0.0892 | Gluconic acid | | | −0.0060 |
| Ketoleucine | | 0.0638 |  | | |  |
| PC(O-34:2) | | 0.0622 |  | | |  |
| *m/z* 219.1140 (ESI−) | | 0.0521 |  | | |  |
| L-Methionine | | 0.0397 |  | | |  |
| *m/z* 175.1244 (ESI−) | | 0.0163 |  | | |  |
| PC36:4(20:3/16:1) | | 0.0125 |  | | |  |

ESI: electrospray ionization; GM3: monosialodihexosylganglioside; *m/z*: mass to charge ratio; PC: phosphatidylcholine; PE: phosphatidylethanolamine; SM: sphingomyelin; TAG: triacylglycerols.
